# Book Reviews

**Published:** 1916-07

**Authors:** 


### BOOK REVIEWS

---

**Books mentioned may be inspected at and ordered through this office. So far as possible, books received in any month will be reviewed in the issue of the second month following.**

---

**The Medical Clinics of Chicago.** Volume I, Number VI, (May 1916). Octavo of 229 pages, 22 illustrations. Philadelphia and London; W. B. Saunders Company, 1916. Published Bi-Monthly. Price per year: Paper, $8.00; Cloth, $12.00.

Hamburger's clinic on the Allen treatment of diabetes contains two points of great practical interest: (1) "While it has been conclusively proved that diabetics even of the severest type may be rendered sugar-free by the Allen method, (2) it will probably take years to determine whether such patients do best in the long run under such conditions." We are disposed to question the first part of this statement, not from personal evidence or opinion but out of deference to the numerous authorities—more numerous in the past than the present,—who have recognized a type of diabetes in which sugar is formed from non-carbohydrate material, remembering that a patient who receives no food is virtually on a diet of fat and proteid. Williamson's clinic on Biliary Hypertrophic Cirrhosis of the Liver is especially interesting because of the rarity of the disease, especially in America. This case occurred in a Russian. Abt's clinic on "rachitis" may not throw new light on this common and poorly understood condition but it ably assembles the various facts. By the way, just why are so many medical writers doing an ectomy on rHachitis? As we understand it no Greek word began with r alone.

---

**Therapeutic By-Ways.** Being a collection of therapeutic measures not to be found in the text books collected from all sources. Condensed and arranged by Dr. E. P. Anshutz. 195 pages. Cloth, $1.00 net. Philadelphia, Boericke & Tafel, 1916.

The author has presented therapeutic suggestions, alphabetically by titles of conditions and diseases, cenceding that some of them are foolish or worthless. We rather question whether an author should be quite so broad-minded but the inclusion of all sorts of therapeutic suggestions certainly adds to the interest of the book and perhaps the puzzled doctor will instinctively glean the rational ones from the chaff. Dosage ranges from the decillionth potency to fair sized amounts of crude drugs and tinctures, infusions, etc.; both external and internal administration of drugs and various devices not requiring drugs are included.

---

**Les Fievres Paratyphoides,** Dr. J. Carles, Bordeaux. Published by J. B. Bailliere et Fils, Paris. 95 pages, 15 illustrations, 1½ francs.

This is one of the series of Actualites Medicales (the word actual in French signifying present or up-to-date). Even before the war but especially since the opportunities which it has afforded for amassing large statistics paratyphoid has become a common and definitely recognized condition. The typhoid bacillus, A and B types of the paratyphoid and the colon bacillus, are thoroughly established and subject to differential diagnosis and differential or combined prophylaxis. To a certain degree, differential diagnosis may be made clinically though it rests mainly on bacterial reactions. This work puts the whole subject on a perfectly definite basis. Whether the differentiations applicable in Europe will be found literally to apply to conditions in America is a question to be settled by further investigation. We strongly advise both laboratory workers and practitioners to read this book in order that we may, in this country, be prepared to recognize in their true relations what are no longer to be considered as mild or atypic cases of typhoid.

---

**Physical Anthropology of the Lenape or Delawares and of the Eastern Indians in General.** Ales Hrdlicka, Bureau of American Ethnology Bulletin 62. 130 pages, illustrated.

Roughly speaking, the aborigines of the eastern half of N. America, up to the region occupied by the Esquimaux are divided into two racial groups, the more northern dolichocephalic (corresponding somewhat with the earlier linguistic groups of Huron-Iroquois and Algonquins) and the more southern brachycephalic, though mutual penetrations of the two races into each other's territory and blending of races are observed. Dr. Hrdlicka's studies constitute a remarkable monograph on osteology with minute descriptions both of racially characteristic points and of individual anomalies. Quite aside from the interest which one may have in American anthropology, the book is well worth examination merely to establish a standard of careful osteologic study.

---

**The Practical Medicine Series** under general editorship of Chas. L. Mix, A. M., M. D., of Chicago. Published by the Year Book Publishers, Chicago. 10 volumes, $10.

Vol. 1, 1916, General Medicine, edited by Frank Billings, M. S., M. D., 384 pages, illustrated, $1.50 separately.

While this series is a review of periodic literature and necessarily discusses subjects as they arise, it seems to us that this volume is more than usually interesting and valuable from the amount of material concerning rarer diseases and those involving ductless glands and other structures, in such ways that pathogeny rests largely on physiology.

---

**Elementary Bacteriology and Protozoölogy.** For the Use of Nurses. By Herbert Fox, M. D., Director of the William Pepper Laboratory of Clinical Medicine in the University of Pennsylvania. Second Edition, Revised and Enlarged. 12mo, 251 pages, with 68 engravings and 5 colored plates. Cloth, $1.75, net. Lea & Febiger, Philadelphia and New York, 1916.

It may, of course, be questioned whether this subject is not too abstruse for the nurse although it may be said that the author has presented it in a very simple and understandable form. We are inclined to believe, however, that aside from any practical considerations, such a book is likely to stimulate the interest of any intelligent nurse in her work. But nursing, especially with the stimulus of existing war in a large part of the world, is becoming more and more a career for highly educated women and even for men of a much higher grade than formerly. There are special fields of nursing, military, municipal, etc., in which bacteriologic investigation is of importance almost as a routine and this routine cannot be successfully carried out except through the active, intelligent co-operation of the nurse. For such duties as these, the author has facilitated preparation in a most skillful handling of his subject.

---

**Pulmonary Tuberculosis.** By Maurice Fishberg, M. D., Clinical Professor of Tuberculosis, University and Bellevue Hospital Medical College; Attending Physician, Montefiore Home and Hospital for Chronic Diseases, New York. Octavo, 639 pages, with 91 engravings and 18 plates. Cloth, $5.00, net. Lea & Febiger, Publishers, Philadelphia and New York, 1916.

This is a highly conservative text book, the author striving to include all known diagnostic and therapeutic measures rather than to advance novel theories. What will appeal strongly to the general practitioner is that the author has subjected every point to the test of actual, extensive experience and that while the clinical studies upon which the work is based are not exclusively among patients in private practice, nearly all cases have been observed under similar conditions. The author lives and finds his clinical material in New York, and, for the greater part of his work, is compelled to deal with his patients under the exactions of city life. We do not mean by this to imply that a work of the same nature written by one in charge of an institution located in an ideal climate and at an ideal altitude and with the advantages of institutional control of diagnostic and therapeutic measures, would be an easy task or without value to the general practitioner. Yet it seems to us that the guidance of one compelled for the most part to care for his patients under the disadvantages of urban life and without special hygienic facilities, is more apt to be applicable to the needs of the general practitioner contending with the difficulties of control of tuberculosis in private practice.

---

**A Treatise on Medical Practice,** Based on the principles and therapeutic applications of the Physical Modes and Methods of Treatment (Non-medicinal Therapy). Otto Juettner, A. M., Sc. M., Ph. D., M. D., of Cincinnati. Published by A. L. Chatterton Co., N. Y. 519 pages. $5.

To prevent a misconception of the author's point of view, it should be stated that he is affiliated with the A. M. A. and various local and national, as well as foreign medical organizations representative of the medical profession in general. He writes, therefore, without sectarian bias and with the proper personal preference for certain forms of treatment as contrasted with others. We do not understand even that he opposes the use of medicinal agents ordinarily so-called but that it is his wish, in this work, to present other means which may be employed by all practitioners, irrespective of such disputes of school or personal opinion as apply to drugs. The work is arranged alphabetically and is, as strictly as possible limited to therapeutics, although in the general discussion of X-rays, water, suggestion, vibration, etc., he has entered into underlying principles. We do not feel either that the reader should neglect medicinal therapy or that the author would wish him to do so but that this concentration of the mind upon non-medicinal measures is highly suggestive and valuable.

---

**The Locust Flower and the Celibate:** Two plays by Pauline Brooks Quinton. Sherman, French & Co., Boston.

We have published several articles on medical subjects by Major W. W. Quinton, M. D., of Buffalo and are, therefore, interested in the literary productions of his wife who has already become known as an author, especially along dramatic lines. Both of these plays, whether considered as potentially staged or as "plays to be read" are of strong emotional interest since they appeal to the intellect and represent psychologic insight. The first play illustrates the paradox that, in impressing a basic truth of psychology, the argument gains strength by reverting to the older, discarded beliefs in spirits as in developing the most powerful and most accurate mathematic principles, we must base our logic on the imaginary and impossible. In this play, there are two real characters, lovers, and "shades of money:" Love of a Boy,

Passion of Youth, Flame of Desire, Heart's Ease, Love of a Man, Lesser Loves—who gave as little as they took and were as soon forgot,—and The Dead. None of the former but only the last shade, of the man's dead and well beloved wife, separate the lovers. The central thought of the play is not weakened by argument or conspicuous attempt to develop a purpose. Nevertheless, it looms up as a question. Why is it that in a civilization whose ideals, at least, are lofty and shaped more than any other past or contemporary civilization by the religious conception of a real, self-conscious immortality, the strongest bond of love should be regarded as a purely temporal state of mind, broken off by death?

The Celibate, whose theme is suggested by Das Hexenlied, is placed in Italy in the fourteenth century. It is a strong contrast of passion and love and, more particularly of the conflict between pure love and monastic vows, developed along unusual lines to an ethical decision which suggests an opportunity for dogmatic controversy. Both of these plays possess the element of human interest in great measure and, as intimated, lead to the consideration of various social and ethical problems so that, after the reader has been entertained, he has something left to think about.

---

**Gynecology.** Wm. P. Graves, A. B., M. D., Boston. W. B. Saunders Co., Philadelphia and London. 770 pages, 303 half tone and pen drawings by the author, 122 microscopic drawings by Margaret Concree and Ruth Huestis, 66 illustrations in color. $7. (Half morocco, $8.50).

The artistic skill of the author enables him to express himself in pictures as most persons can do only in words. Thus, he has been able to emphasize details in a double way and to convey his exact ideas. Anyone who has attempted to make a professional artist convey his meaning can appreciate the great advantage possessed by an author who can draw. Look carefully at the average landscape: you see trees and plants, but usually no particular kind of tree or herb. Perhaps you can make out that a tree is a willow or a plant, a rose, but you will be unable to say what species and the botanist can usually detect palpable errors. The need of technical accuracy in an illustration of a pathologic condition or of a surgical operation is great. Wisely, we think, the author has taken for granted a knowledge of anatomy. The physiology of the uterus and ovary are discussed somewhat in detail. There follow about 90 pages of scholarly discussion of the relationship of gynecology to the general organism, the ductless glands, mammary glands, skin, organs of sense, blood, bones and joints, etc., being considered in detail while special headings are made of acute infections, enteroptosis, movable kidney and intestinal bands. To the best of our recollection, no other author has assembled in a text book, so complete a discussion of this kind. The second part of the book, entitled Gynecologic Diseases, is not so like "medical gynecology" as gynecology pathology, the microscopic drawings appearing especially in this part. However, it would be unjust to leave the impression that diagnosis and treatment are neglected. The third part, Operative Gynecology, more closely resembles the prevailing type of text book on gynecology.

---

**A Text-Book of Fractures and Dislocations, With Special Reference to Their Pathology, Diagnosis and Treatment.** By Kellogg Speed, S. B., M. D., F. A. C. S., Associate in Surgery, Northwestern University Medical School; Associate Surgeon, Mercy Hospital; Attending Surgeon, Cook County and Provident Hospitals, Chicago, Ill. Octavo, 888 page, with 656 engravings. Cloth, $6.00, net. Lea & Febiger, Publishers, Philadelphia and New York, 1916.

Briefly but thoroughly, the author summarizes the opinions as to processes of repair often without expressing a personal opinion but leaving the reader in no doubt as to facts definitely established. The mechanics of fractures and dislocations and the general principles of diagnosis and treatment follow, making up about a fifth of the book. From this point, fractures and dislocations are considered in detail, the latter being described after the fracture of a bone, as nearly as possible according to anatomic location. At first thought, it might seem better to separate fractures and dislocations entirely since the latter cannot, of course, be classified by bones but the frequent association of the two pathologic conditions, the similarity in symptomatology and in the mechanic principles involved in holding a given region in its normal alignment, whether the obstacle is a fracture of an osseous unit or a dislocation of the junction of two such units, are very practical reasons for following the order chosen. While the book makes no special appeal on account of "timeliness"—a much over-worked argument for all kinds of literature—and is a systematic treatise on the whole subject, bone grafting and various other modern methods and devices are well treated.

---

**Embryology, Anatomy, and Diseases of the Umbilicus Together With Diseases of the Urachus.** By Thomas S. Cullen, Associate Professor of Gynecology in the Johns Hopkins University. Large octavo of 680 pages with 269 original illustrations and 7 plates by Max Brodel and August Horn.

Philadelphia and London: W. B. Saunders Company, 1916. Cloth, $7.50, net; Half Morocco, $9.00, net.

Many a true word is spoken in jest. It was long ago said that the development of specialism would leave nothing for an original choice but the umbilicus; or that this would be all that was left for the general practitioner. The author has not only shown that a vast amount of research can be centered in the umbilicus but he has pursued his research and directed his practical investigation in such a way as to demonstrate that the general practitioner can by no means neglect this small part of the body for the practical problems radiating from it are numerous and important. The embryology of the umbilical region is illustrated with numerous colored plates. 35 pages of descriptive anatomy follow, with photographic reproductions of various types. Infections, haemorrhages and granulation tissue are then discussed, Meckel's diverticulum, intestinal cysts, tumors, herniae, escape of round worms, calculus formation, etc., follow. The urachus including exstrophy of the bladder, urinary fistulae and other thoroughly practical subjects, takes up 175 pages. For each chapter, bibliographic references are given and there is a final index of authorities beside the general index. We feel that this American book may be compared favorably with the highest type of German concentration while, at the same time, it exemplifies the broadness of view and the practicality more characteristic of French and American works.

---

**The Art of Anaesthesia.** Paluel J. Flagg, M. D., N. Y., J. B. Lippincott Co., Philadelphia. 341 pages. $3.50.

Historic references are given to the use of narcotic drugs in the pre-anaesthetic period and fac-simile letters referring to "ether frolics" and the earliest use of ether for an operation, by Dr. C. W. Long in 1842. General and local anaesthesia, the use of instrumental aids and the entire art of the anesthetist are fully described.

---

**Blood-Pressure: Its Clinical Application.** Second Edition, Revised and Enlarged. By George W. Norris, A. B., M. D., Assistant Professor of Medicine in the University of Pennsylvania; Visiting Physician to the Pennsylvania Hospital; Assistant Visiting Physician to the University Hospital; Fellow of the College of Physicians of Philadelphia. Octavo, 424 pages, with 102 engravings and 1 colored plate. Cloth, $3.00, net. Lea & Febiger, Publishers, Philadelphia and New York, 1916.

The physiologic forces concerned in maintaining blood pressure are stated to be the cardiac systole, the arterial elasticity, the arterioles and capillaries, which afford most of the resistance to the two former factors, the veins and capillaries, forming a reservoir with just enough pressure to fill the relaxing heart promptly, the blood itself, as a practically incompressible medium, and the lymphatic system with its tissue spaces and cavities. Variations in pulse provided the heart has time to fill itself, do not appreciably affect blood pressure. A great many interesting physiologic details are given, which should be thoroughly studied. Instruments and technic follow the physiologic discussion, completing approximately the first half of the volume. Essential hypo—and hypertension, and the relation of various diseases and conditions of strain, shock, etc., to blood pressure are then considered seriatim. One chapter is devoted entirely to the effect of drugs and glandular extracts on blood pressure, with special consideration of the coronary vessels and venous pressure. The bearing of blood pressure on life insurance is fully discussed.

